# Central Serous Chorioretinopathy and Personality Characteristics: A Systematic Review of Scientific Evidence over the Last 10 Years (2010 to 2020)

**DOI:** 10.3390/medicina57060628

**Published:** 2021-06-16

**Authors:** Giovanni Genovese, Alessandro Meduri, Maria Rosaria Anna Muscatello, Sebastiano Gangemi, Clemente Cedro, Antonio Bruno, Pasquale Aragona, Gianluca Pandolfo

**Affiliations:** 1 Department of Biomedical and Dental Sciences, Morphological and Functional Images, University of Messina, 98121 Messina, Italy; alessandro.meduri@unime.it (A.M.); mmuscatello@unime.it (M.R.A.M.); clemente.cedro@unime.it (C.C.); antonio.bruno@unime.it (A.B.); pasquale.aragona@unime.it (P.A.); gpandolfo@unime.it (G.P.); 2 School and Operative Unit of Allergy and Clinical Immunology, Policlinico “G. Martino”, Department of Clinical and Experimental Medicine, University of Messina, 98125 Messina, Italy; gangemis@unime.it

**Keywords:** central serous chorioretinopathy, personality, temperament, character, psychiatry

## Abstract

*Background and Objectives*: to investigate the current state of art in the study of personality disorders in central serous chorioretinopathy (CSC), also taking into account the dimensional approach. *Materials and Methods*: this systematic review was conducted according to PRISMA guidelines. We included articles written in English or Italian, published in peer reviewed journals from 1 January 2010 to 31 December 2020. *Results*: after the screening, 10 studies were included. The results suggest that CSC patients are not characterized by the prevalence of a formal personality disorder, but they are better explained by typical personality traits that may alter their relationship with others. CSC patients seems to be characterized by high levels of aggressiveness and anxiety traits along with low sociability. We propose a model of disease where stress exacerbates prior specific traits in a vicious circle where some traits might be involved in disease progression and manifestation. *Conclusions*: maladaptive personality traits might be an essential feature of the disease and may represent a possible link between psychiatric symptoms, such as insomnia, anxiety, and depression, and endocrinological patterns. Further research should use a specific assessment scale evaluating both the level of interpersonal functioning and specific maladaptive traits.

## 1. Introduction

Central serous chorioretinopathy (CSC) is a common pachychoroid spectrum disorder (PSD) characterized by the presence of a thick choroid associated with increased vascular permeability and serous detachment of the neurosensory retina. PSD includes different expressions of a continuous process with several degrees of impairment, where CSC is considered the archetypical event [1]. CSC typically affects middle-age males, and it is characterized by sudden onset and spontaneous resolution of symptoms within three months [2]; in a smaller percentage of cases, the disorder can evolve in a continuous or recurrent course [3]. It is known that increased capillary permeability is one of the major features of CSC, and several important biochemical factors were reported to be involved in the pathophysiology of CSC [4,5]. Although CSC pathogenesis is far from being fully understood, other well-established associations are endocrinological factors, psychological distress [6,7], and psychiatric conditions such as anxiety, depression [8,9], and sleep disorders [10]. Psychopharmacological therapy might also be associated to CSC [11]: exposure to anti-anxiety drugs within a one-year period before CSC onset was independently associated with idiopathic CSC among males only [12], whereas the use of a psychiatric medication at presentation was protective for vision loss [13]. Among endocrinological factors, in CSC patients, several lines of evidence highlight alterations in the hypothalamic–pituitary–adrenal (HPA) axis, which is involved in stress response, diurnal cycle, and some psychiatric diseases such as post-traumatic stress disorder [14] and major depressive disorder [15]. Moreover, exogenous corticosteroid administration may precipitate the disease [16]. A long scientific tradition attributes the type-A personality, a pattern of behavior characterized by aggressiveness, hostility, competitive drive, and a sense of time urgency [17], as prevalent in central serous chorioretinopathy (CSC) patients. Since Schneider reported psychopathic personalities in the 1920s, psychiatrists have catagorized abnormal personality characteristics into categorical patterns, such as the well-known Axis II of the Diagnostic and Statistical Manual of Mental Disorders III [18]. Researchers in recent years have sparked a heated debate about nosology in personality disorders that culminated in the integration of the dimensional approach, alternative to the categorical approach, into the Diagnostic and Statistical Manual of Mental Disorders, 5th edition [19]. The dimensional model describes the presence of 25 maladaptive personality traits, organized into five domains. The diagnosis of personality disorders requires the fulfillment of other criteria which account for the level of personality functioning, pervasiveness, stability, discrimination from other mental disorders, effects of substances or medical conditions, and the developmental stage or sociocultural environment.

A chronic visual disease highly compromises quality of life [20,21]; therefore, the identification of risk factors is crucial for early treatment and intervention. An important line of research about CSC is currently focused on differences between patients with an acute disorder and those who develop a chronic disorder [22,23,24]. Emerging evidence highlights that patients with non-organic sleep disorders have a higher incidence of CSC than controls, independent of any other comorbidity [25]. Personality disorders and insomnia show a mutual influence within a circular model in which insomnia is related to maladaptive personality traits and, on the other hand, personality disorders promote the onset of insomnia [26]. It is likely that some personality dimensions are among the risk factors of disease progression, potentially suitable as targets for early interventions. In this context, our review aims to evaluate the possible role of personality traits and disorders in the onset, course and prognosis of CSC, also taking into account the dimensional approach towards personality disorders implemented by current studies. The second aim of the present review is to investigate the role of early treatments focused on specific personality dimensions for the prognosis of CSC in subjects at risk of chronicization.

## 2. Materials and Methods

### 2.1. Search Processes

This systematic review was conducted according to PRISMA (Preferred Reporting Items for Systematic Reviews and Meta-Analyses) guidelines [27]. The PubMed database was searched until 31 December 2020, using the following key terms: “central serous chorioretinopathy”, “mental disorder”, “personality”, “temperament”, “character”, “psychiatry”. The electronic search strategy used for PubMed is described in Table A1 (Appendix A). Articles were selected by title and abstract; the full article was read if the title and abstract was related to the specific issue of CSC and personality, and if the article potentially met the inclusion criteria. References of the selected articles were also examined in order to identify additional studies meeting the inclusion criteria (Table 1).

### 2.2. Study Selection

Articles were included in the review according to the following inclusion criteria: written English or Italian, published in peer-reviewed journals or books, and quantitative and qualitative information on CSC and personality. In order to focus on the latest evidence, we selected publications from 1 January 2010 to 31 December 2020. Articles were excluded by title, abstract or full text for irrelevance to the topic in question. Further exclusion criteria were articles published before 1 January 2010, not written in English or Italian, unpublished dissertations and theses, and other non-peer-reviewed material.

### 2.3. Data Extraction

Two authors (G.P., G.G.) performed the initial search and independently reviewed and selected the references based on the inclusion and exclusion criteria. The results were subsequently re-evaluated by the auditors and the salient results were shown. Data derived from our research of articles included the study author names, publication dates, study designs (i.e., open-label uncontrolled and randomized controlled trial), samples (case and control group), tests used for the assessment, and main findings.

## 3. Results

**Figure 1:**
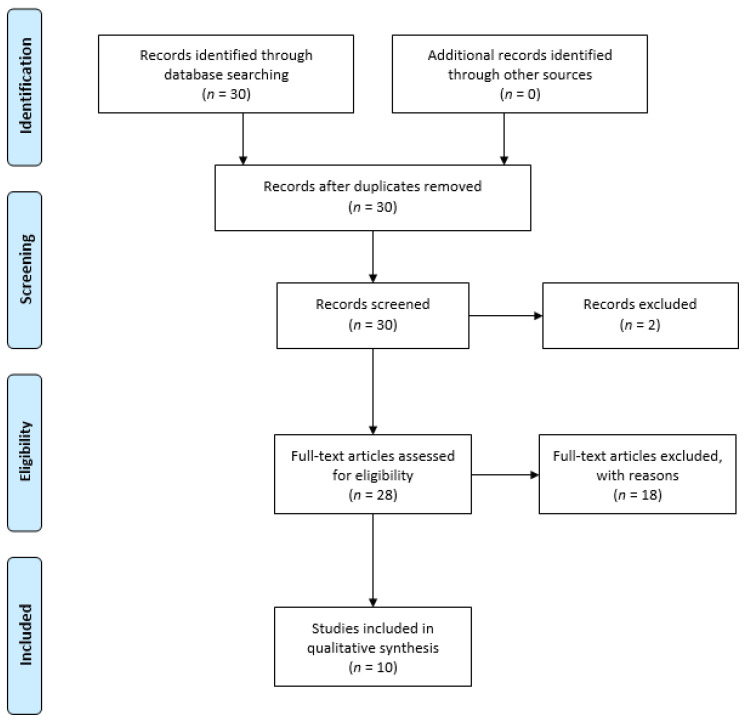
Flow diagram of the literature selection process.

We found 30 research papers that evaluated personality factors in CSC patients. Of these, 18 studies were excluded because they were irrelevant to the topic, one study was excluded because the full text was written in Polish, and one study was excluded because the full text was not available. After the screening, 10 studies assessing personality met the inclusion criteria and were included in the systematic review. Figure 1 summarizes the flow chart of articles selected for the review. Table 1 summarizes the selected articles.

Four studies describe a higher prevalence of type-A personality in CSC patients [29,31,32,33], although wide differences between data emerged (96.8% of patients who recognized precipitating factor in Romano et al. 2019 [29]; 26.19% in Islam et al. 2017 [33]). Mansour et al. (2017) [31], in addition to type-A personality, evaluated personality traits in CSC patients not affected by chronic, non-resolving disease. All patients were assessed using a psychodiagnostics battery composed of different tests in a short form. The CSC group showed higher obsessive-compulsive and aggressive behaviors. Statistically significant differences between groups were observed in several other dimensions, such as distress, work devotion, sleep disturbance, a history of panic attacks or premature ejaculation, and the use of psychopharmacologic drugs. In contrast, van Haalen et al. (2019) [30] focused on personality traits in chronic CSC patients with active or inactive disease. Patients with other retinal disease and those who used corticosteroids or sleep medications were excluded from the study. The results showed that 20.9% of patients with chronic CSC had a history of psychiatric disorders (depression, anxiety or panic disorder, post-traumatic stress disorder, burnout, alcohol abuse and schizophrenia). Patients with CSC did not report more apathy or irritability compared with general population. Numerous other personality dimensions, such as submissiveness, cognitive distortion, affective lability, stimulus seeking, compulsivity, oppositionality, anxiousness, suspiciousness, social avoidance and insecure attachment were lower than those from the general population. Patients with inactive CSC showed more affective lability, submissiveness and social avoidance compared to patients with active disease. Compared with the general population, patients with CSC showed more intimacy problems and, less narcissism. Carlesimo et al. (2014) [35] described a case of CSC in a 43-year-old male patient affected by narcissistic personality disorder as assessed by Minnesota multiphasic personality inventory (MMPI); the MMPI further showed a tendency toward somatization. Other studies have been performed to evaluate personality traits or dimensions in CSC patients. Mylona et al. (2020) [28] showed that CSC patients’ scores, compared with healthy controls (HC) and patients with non-CSC ophthalmic diseases, were significantly higher on three of the five dimensions investigated: neuroticism/anxiety, aggression/hostility, and activity, whereas they scored significantly lower in sociability than the other two groups, and in impulsivity than non-CSC patients. Lahousen et al. (2016) [34] evaluated personality traits in a sample of CSC patients compared with patients affected by other ophthalmic diseases. Exclusion criteria included the presence of psychiatric symptoms. Patients with CSC reported higher levels of personality traits such as impulsiveness, aggressiveness, strain, frankness, and emotionality, along with psychosomatic symptoms and rumination than controls. Interestingly, higher scores of aggressiveness were found in chronic CSC subjects when compared with patients affected by acute CSC. Two studies [36,37] evaluated temperament and character features associated with CSC by using the temperament and character inventory (TCI). Piskunowicz et al.’s (2014) [36] main results were that CSC patients scored higher on harm avoidance (HA) and reward dependence (RD), and lower on several novelty seeking (NS) subscales than controls. They suggested that the examined CSC patients had a personality profile mainly characterized by ineffective stress coping strategies, the tendency to avoid negative and potentially harmful stimuli, insecurity, and anticipatory anxiety along with less exploratory behaviors and restraint. Conrad et al. (2014) [37] found that the CSC group scored lower than controls on cooperativeness, a character dimension, and reward dependence, a temperamental trait. TCI dimensions in this work were associated with illness-related work stress.

## 4. Discussion

Starting from Yannuzzi (1986) [38], to date, the construct of the type-A personality has gained widespread consensus among researchers focused on CSC, due to its hypothesized association with higher levels of circulating catecholamines and corticosteroids. Recent research is going beyond the concept of type-A personality in CSC, giving a multifaceted view of the personality characteristics in these patients. However, differences in methods, samples, and assessments make the available data scarcely comparable. Only two studies [36,37] shared the same assessment instrument, the TCI, although providing conflicting results. The temperamental profile found in the small group of CSC patients from Piskunowicz et al. (2014) [36] was comparable with the type-D personality, which is characterized by a blend of negative affectivity, social inhibition and vulnerability to stress; this result is quite contrasting with the available data on the prevalence of type-A personality in CSC patients. An interesting speculation could emerge from this data, since negative affectivity and social inhibition, two main dimensions of type-D personality, were independently associated with insomnia [39], a well-known risk factor for CSC. Quite differently, Conrad et al. (2014) [37] found low levels of cooperativeness, which is considered an almost reliable marker for the presence of a personality disorder [40]. Furthermore, Conrad et al. (2014) [37] pointed out analogies between CSC patients and Machiavellian and antisocial personality traits. This last assumption advances several options for discussion, since impulsivity, a nuclear feature of antisocial personality, has not been congruently reported in CSC patients [28,34], thus suggesting that this is not a core characteristic of the disease. Furthermore, the documented tendency by CSC patients to seek social support [30] is quite incompatible with the presence of Machiavellian and antisocial traits. Regarding narcissistic traits described by Carlesimo et al. (2014) [35], this association coming from a single case report has not been confirmed by further data, as those from van Haalen et al. (2019) [30] showed that CSC patients obtained lower scores on the DAPPsf’s narcissistic subscale than controls.

According to the available data about personality traits, CSC patients should be characterized by high levels of aggressiveness and anxiety traits and by low sociability [28,30,31,37,41]. Aggressiveness appears to be a recognizable feature even in CSC patients without manifest psychiatric symptoms, with higher levels in the chronic CSC subtype than in the acute one [34]. CSC does not seem to affect the level of activity and it is not associated with apathy or irritability [28,30]. Other emerging evidence points at obsessive-compulsive behaviors [31] and rumination [34]. Furthermore, van Haalen et al. (2019) [30] found more affective lability and social avoidance in patients with inactive CSC compared to patients with active disease [30].

**Figure 2:**
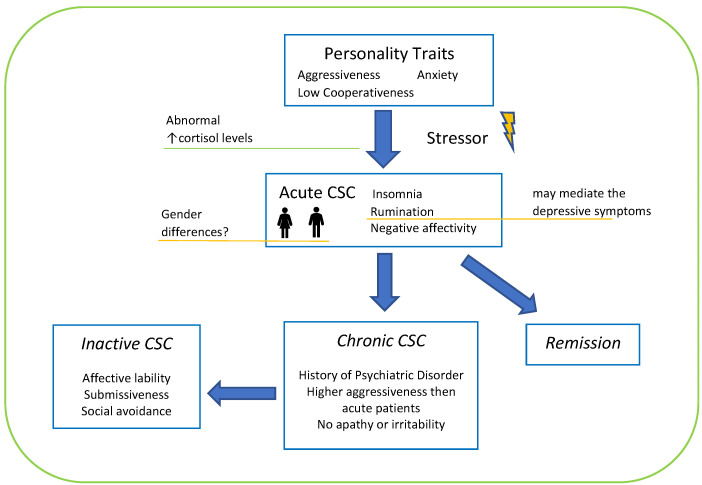
Personality traits and phase of disease. Orange: need further investigation.

These results suggest that CSC patients are not characterized by the prevalence of a formal personality disorder, but they are better explained by a blend of personality traits that may alter their relationship with others, especially in emotionally charged contexts. According to our understanding (Figure 2), stress might exacerbate the above-mentioned characteristic traits and co-occurrence of insomnia, followed by visual loss. Visual impairment makes patients seek more support from others, and stressful socio-relational interaction, correlated with dysfunctional traits, may arouse abnormal anxiety levels, along with manifest CSC symptoms. In a speculative view, the presence of ruminative tendencies, identified in one paper [34], may mediate the development of depressive symptoms documented in a proportion of patients [9]. High levels of aggressiveness, together with negative affectivity and social inhibition, may be responsible for social and relational conflicts, increasing subjective distress in a vicious circle that could potentially contribute to the progression of the disease, with psychopathological alterations detectable both in active and inactive chronic CSC patients. However, it should also be considered that the HPA axis alterations in response to stressful situations are well known in CSC patients, and cortisol levels were found to be correlated with personality alterations, taking into account gender differences, since in male subjects a direct correlation between cortisol levels and neuroticism was found, whereas in females the same relationship was inverse [28,42]. These findings may partly explain the higher prevalence of CSC in males [43], and trace a mutual correlation between CSC and personality. None of the studies considered gender differences and personality traits in CSC patients despite a relevant portion of the sample being composed of female patients (from 10% to 28%).

## 5. Conclusions

Current studies in the field of CSC show that there are widely unmet needs that further research should fulfill. Personality has not yet been fully investigated in its dimensional and trait models, and the available data cannot be included in coherent and shared theoretical constructs, nor can it add further knowledge within this field of research, due to the marked differences in methods and to the small samples. Furthermore, only a few studies have evaluated the influence of personality traits on CSC course and recurrence, and, in accordance with Mylona et al. (2020) [28], our data support the hypothesis that further research is needed to clarify gender differences in CSC patients. Identifying specific personological dimensions related to levels of functioning might be of great interest for adding new insight into both CSC pathophysiology and mechanisms of chronicization. Maladaptive personality traits might be an essential feature of the disease and they may represent a possible link between psychiatric symptoms, such as insomnia, anxiety and depression, and endocrinological patterns. Further research should use a specific assessment scale; we propose the DSM 5 alternative model for personality disorders as a suitable candidate for its integrative nature, evaluating both the level of interpersonal functioning and specific maladaptive traits, derived from empirical research [44]. Last but not least, it would be interesting to compare typical personality traits detectable in CSC patients compared to other pachychoroid spectrum disorders.

## Figures and Tables

**Table 1:** The table recapitulate the manuscripts evaluating personality Central serous chorioretinopathy (CSC) patients.

Reference	Subjects	Assessment	Main Findings
Mylona I. et al. (2020) [28]	100 CSC patients (79 male), 200 healthy control (HC), 200 non-CSC patients.	The Zuckerman–Kuhlman personality questionnaire (ZKPQ)	CSC patients, compared to the other groups, scored significantly higher on neuroticism/anxiety, aggression/hostility, and activity; they scored significantly lower in impulsivity compared to non-CSC patients.
Romano M.R. et al. (2019) [29]	1719 CSC patients (1326 male, 77.1%)	The questionnaire asked patients about whether they were considered to have a high-stress personality	A total of 33.2% of patients recognised contributing factors (60.1% unknown and 6.7% missing); 96.8% of that 33.2% reported having a type-A personality
van Haalen F.M. et al. (2019) [30]	86 chronic CSC patients (77 male, 90%)	Dimensional assessment of personality pathology short form (DAPPsf); The apathy scale (AS); the irritability scale (IS); The Utrecht coping scale (UCS)	Patients with CSC did not report more apathy or irritability compared with the general population at the AS and IS. The authors did not find a higher prevalence of maladaptive personality traits in patients with CSC compared with the general population.
Mansour A.M. et al. (2017) [31]	86 CSC patients (67 male, 80.7%) and 86 non-CSC patients.	They administered an interview composed of different tests in a short form.	CSC group compared to controls showed higher obsessive-compulsive and aggressive behaviors, and higher type-A personality traits
Chatziralli I. et al. (2017) [32]	183 first episode CSC patients (131 male, 71.6%); 183 HC.	Jenkins activity survey	Type-A personality and stress were associated with CSC.
Islam Q.U. et al. (2016) [33]	42 acute CSC (38 male, 90.47%).	NA	A total of 35.71% of CSC patients suffer from emotional stress or psychiatric disorder; 26.19% have in a type-A personality.
Lahousen T. et al. (2016) [34]	95 CSC patients (37 acute, 49 chronic; 71 male, 74.7%); 75 other ophthalmic patients.	The questionnaire to critical life events; Stressverarbeitungsfragebogen (SVF 120); Freiburg personality inventory (FPI-R).	Patients with CSC reported higher results then controls in psychosomatic symptoms, rumination, and several personality traits. The chronic CSC group showed higher scores in aggressiveness than the acute CSC subtype.
Carlesimo S.C. et al. (2014) [35]	One Male	Minnesota multiphasic personality inventory (MMPI)	A narcissistic personality disorder was diagnosed after CSC onset. The MMPI showed a tendency towards somatization.
Piskunowicz M. et al. (2014) [36]	32 acute CSC (27 male, 84%), 30 HC	The temperament and character inventory (TCI)	CSC patients showed higher scores than controls in harm avoidance and reward dependence but lower scores in subscale sentimentality, as well as lower scores in novelty seeking but higher score in subscale disorderliness.
Conrad R. et al. (2014) [37]	57 CSC patients (45 male, 78.9%) 57 HC	The Symptom Checklist 90-Revised (SCL-90-R); the TCI; the global severity index (GSI)	The CSC group compared to HC showed significantly higher results on GSI and SCL-90-R. CSC group was associated with a significantly lower score than controls on cooperativeness and reward dependence. Both these TCI dimensions and the subjective assessment of severity of illness correlates with illness-related work stress.

## Data Availability

Not applicable.

## Appendix A

**Table A1:** List of search terms entered into the PubMed search engines in order to identify the articles for this systematic review.

Number	Search Term
1	“Central serous chorioretinopathy”
2	“Mental disorder”
3	“personality”
4	“temperament”
5	“character”
6	“psychiatr*”
7	1 AND 2 OR 3 OR 4 OR 5 OR 6
8	English OR Italian [Language]
9	From 1 January 2010 to 31 December 2020
